# The complete mitochondrial genome and phylogenetic placement of *Apis nigrocincta* Smith (Insecta: Hymenoptera: Apidae), an Asian, cavity-nesting honey bee

**DOI:** 10.1080/23802359.2017.1318683

**Published:** 2017-04-25

**Authors:** Amin Eimanifar, Rebecca T. Kimball, Edward L. Braun, Stefan Fuchs, Bernd Grünewald, James D. Ellis

**Affiliations:** a Honey Bee Research and Extension Laboratory, Entomology and Nematology Department, University of Florida, Gainesville, FL, USA;; b Department of Biology, University of Florida, Gainesville, FL, USA;; c Institut für Bienenkunde, Polytechnische Gesellschaft, Goethe-Universität Frankfurt am Main, Oberursel, Germany

**Keywords:** *Apis nigrocincta*, mitogenome, next-generation sequencing

## Abstract

The complete mitochondrial genome of *Apis nigrocincta* was sequenced. The mitochondrial genome is a circular molecule of 15,855 bp, including 37 classical eukaryotic mitochondrial regions and an A + T-rich region. Gene directions and arrangements are similar to those of other *Apis* mitogenomes. Most genes initiated with ATT, though ATG and ATA were also used as start codons. Twelve of 13 protein-coding genes terminated with TAA, though ND2 terminated with TAG. Four PCG genes, eight tRNAs and both rRNAs were encoded on the heavy strand while all others were encoded on the light strand (9 PCGs and 14 tRNAs). Overall, the GC content composed 15.6% of the mitogenome. All of the 22 tRNA genes, ranging from 66 to 114 bp, have a typical cloverleaf structure. A phylogenetic tree showed that *A. nigrocincta* clustered closest to *A. cerana*. The complete mitogenome of *A. nigrocincta* provides essential information on the biogeography and evolution of this Asian honey bee species.

*Apis nigrocincta* F. Smith (1861) is an Asian cavity-nesting species of honey bee, distributed in western Sulawesi, Mindanao Island and on Sangihe Island (Damus & Otis 1997). The vast majority of research on the mitochondrial genome diversity of Asian honey bees has focused on *A. cerana* (Hepburn & Radloff 2011). Only limited mitochondrial data has been published for *A. nigrocincta* and very few studies have been conducted to examine the phylogenetic placement of this species (Arias & Sheppard 2005). Here, we report the complete mitochondrial genome of *A. nigrocincta* (GenBank accession no. KY799147) which has not been sequenced previously.

An adult worker honey bee of *A. nigrocincta* was obtained from the Ruttner Bee Collection at the Bee Research Institute in Oberursel, Germany (Voucher no. 2586, Indonesia, Sulawesi, S. Hadisoesilo, 0°40S, 119°44E). The sample identity was confirmed by institute staff via morphometric evaluation. We extracted and quantified genomic DNA from the thorax of the bee as described in Eimanifar et al. (2016). In short, a genomic library was constructed from the genomic DNA using a Kapa Hyper Prep Kit (Kapa Biosystems, Woburn, MA) with a paired-end read (2 × 150) followed by next-generation sequencing on the Illumina Hi-Seq 3000/4000 (San Diego, CA).

The sequencing reads were trimmed using Trimmomatic v0.35 (Bolger et al. 2014) and mapped to the reference *A.m. ligustica* honey bee mitogenome (L06178.1, the Italian honey bee) using bowtie v2.2.9 (Langmead & Salzberg 2012). The subset of reads was then mapped to the reference using breseq v 0.28.1 (Deatherage & Barrick 2014) to verify that the coverage was correct and uniform. The resulting reads were adjusted and assembled using Spades v3.9.0 (Bankevich et al. 2012). The resulting contigs were blasted against the reference sequence using NCBI blast package v2.2.19 and the best contigs were identified. These contigs then were used as the reference for breseq mapping as a final step to verify that the coverage was even and met. The complete mitogenome of *A. nigrocincta* was 15,855 bp in length, with 13 protein-coding genes (PCGs), 22 transfer RNAs (tRNAs), 2 ribosomal RNAs (rRNAs), and a putative control region. Its overall base composition was 42% (A), 9.3% (C), 6.2% (G), and 42.4% (T). The gene organization, structure, and arrangement were similar to other published *Apis* mitogenomes (Eimanifar et al. 2016).

Four genes, ND1, ND4, ND4L, and ND5, eight tRNAs and both rRNAs were encoded on the heavy strand, while the remaining nine PCGs and 14 tRNAs were encoded on the light strand. Nine PCGs began with an ATT start codon, while ATP6, COIII, and CYB began with ATG, and ND4 began with ATA. All genes shared the stop codon TAA. The longest PCG was ND5 (1668 bp) and the shortest was ATP8 (162 bp). Twenty-two tRNA genes were identified between the rRNA and PCGs, ranging in size from 59 to 77 bp. All tRNAs folded into a typical cloverleaf-shaped secondary structure as identified by tRNAscan-SE (Lowe & Eddy 1997). The sizes of the small ribosomal RNA (12S rRNA) and large ribosomal RNA (16S rRNA) genes were 782 and 1331 bp, respectively.

The phylogenetic position of *A. nigrocincta* with inclusion of 6 other *Apis* species was estimated using RaxML 8.0.20 (Stamatakis 2006) with 1000 bootstrap replicates using 13 PCGs and two rRNAs. *Apis nigrocincta* clustered with *A. cerana* with high bootstrap support (Figure 1). The phylogenetic analysis was consistent with morphological and molecular evidence, indicating that *A. nigrocincta* has similarities with *A. cerana* (Hadisoesilo & Otis 1996; Raffiudin & Crozier 2007).

**Figure 1.**
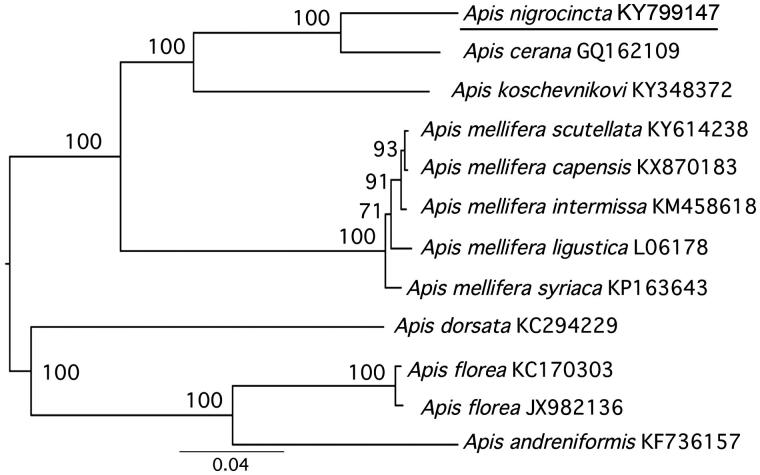
Molecular phylogeny of *A. nigrocincta* based on concatenated dataset (13 PCGs +2 rRNA genes). The phylogenetic tree is constructed with the Maximum Likelihood approach. The GTR + G model was applied to each partition. Eleven mitogenome sequences were obtained from GenBank and included in the tree with their accession numbers. The bootstrap support values are shown next to nodes. The GenBank accession numbers are indicated after the scientific name.

The maximum *p*-distance was between *A. nigrocincta* and *A. andreniformis* (0.16) and the minimum between *A. nigrocincta* and *A. cerana* (0.07). In conclusion, the complete mitogenome of *A. nigrocincta* provides essential and important molecular data for understanding the evolution and biogeography of *Apis*.
